# The Prevalence of Hospitalized Parkinson’s Disease Patients in All Case Hospitalization among Different Race/Ethnic Subgroups in Hawaii

**DOI:** 10.3233/JPD-230341

**Published:** 2024-06-04

**Authors:** Michiko Kimura Bruno, Masako Matsunaga, Emma Krening, Fay Gao, John J. Chen, Todd Seto, G. Webster Ross

**Affiliations:** a The Queen’s Medical Center, Honolulu, HI, USA; b University of Hawaii John A, Burns School of Medicine, Honolulu, HI, USA; c Pacific Health Research and Education Institute, VA Pacific Islands Health CareSystem, Honolulu, HI, USA

**Keywords:** Parkinson’s disease, hospitalization, Native Hawaiian or Other Pacific Islander, Asian, prevalence

## Abstract

**Background:**

Little is known about the epidemiology of Parkinson’s disease (PD) patients in Native Hawaiian Or Other Pacific Islander (NHPI) and Asian American (AA) subgroups.

**Objective:**

To determine if the prevalence of hospitalized PD patients is different across age groups and racial/ethnic subgroups in Hawaii.

**Methods:**

We conducted a retrospective analysis of Hawaii statewide registry (2016–2020) hospitalization data for patients who were 50 years or older. PD patients were identified using an ICD 10 code: Parkinson’s Disease (G20) as their primary/secondary hospitalization discharge diagnosis code. Demographic and clinical characteristics among racial/ethnic subgroups (White, Japanese, Filipino, Chinese, NHPI, or Other) were compared.

**Results:**

Of 146,844 total hospitalized patients (*n* = 429,879 records), 1.6% (*n* = 2,401) had a PD diagnosis. The prevalence of hospitalized PD patients was 2.3% among Japanese and Chinese, followed by 1.7% for Whites, 1.2% for Filipinos and was lowest for NHPI with 0.9% (*p* < 0.001). As patient’s age increased, the prevalence of hospitalized PD patients increased, with 80–84 years old for the highest age range (3.4%). The prevalence of hospitalized PD patients at 80–84 years old varied across the race/ethnic subgroups (Chinese 4.3%, Japanese 4.0%, Whites 3.7%, Filipinos 2.5%, NHPI 2.3%).

**Conclusions:**

The prevalence of hospitalized PD patients among all case hospitalizations were lower for NHPI and Filipino compared to that of Japanese, Chinese, and Whites. As patients’ age increased, the prevalence of hospitalized patients with PD increased, but less so in NHPI and Filipino groups. Further research is warranted to understand the reason for these observed differences among racial/ethnic subgroups.

## INTRODUCTION

The majority of studies documenting racial/ethnic disparities of Parkinson’s disease (PD) in the United States (US) have focused on African Americans and Latinos [1, 2]. Very little is known about the prevalence, correlates, and consequences of PD in Native Hawaiian Or Other Pacific Islander (NHPI) and many Asian American (AA) subgroups, despite NHPI and AA being among the fastest growing racial groups in the US [3]. Previous epidemiological studies suggest that incidence of PD maybe lower among Asians than those of Whites. PD generally has a male predominance, but the degree maybe less in Asians [4]. A small study conducted in Hawaii using drug-treatment and other claims history showed age non-adjusted prevalence of PD in Hawaii to be 145/100,000. The male to female ratio was 1.4 to 1 and 58% of the patients were Japanese [5].

NHPI face numerous socioeconomic disparities, including higher proportion of poverty, higher rates of unemployment, and higher percentage of homelessness [6, 7], which may contribute to health disparities. The limited available data shows that NHPI have significantly higher cardiovascular disease burden, higher overall health burden, and poorer health outcomes than other race groups in Hawaii and the U.S. population as a whole [8–10]. Historically, due to insufficient sample sizes or inadequate data capture, NHPI and AA have been aggregated into a single racial category, that can mask substantial differences among NHPI and AA subgroups [11]. AAs are typically aggregated into one racial group but there are diverse subgroups. While some AA subgroups have higher levels of educational and socioeconomic status, a significant portion of AA do not enjoy these benefits [11] and health disparities have been identified within AA subgroups. Filipinos have been identified as having higher cardiovascular comorbidities in previous studies [12, 13]. Holland, in 2012, recommended collecting and reporting race/ethnicity data by AA subgroups when possible, and also recommended acknowledging that significant heterogeneity exists among AA subgroups when interpreting data [14].

While PD is predominantly managed in ambulatory setting, complications of PD and medical comorbidities, can lead to emergency room (ER) visits and hospitalizations [15, 16]. Underserved minority groups receive less neurologic outpatient care [17] but present to the hospital when they develop illnesses requiring emergent and acute hospital care: thus providing an opportunity to capture them even if they do not consistently seek outpatient care. Aggregate data collected through inpatient admissions can be utilized to bank and analyze valuable information on a large scale.

In the state of Hawaii, the statewide hospitalization registry differentiates NHPI from AA and categorizes AA into subgroups, unlike hospitalization data from most other states, providing a valuable tool to evaluate racial/ethnic disparities. Several studies examining racial/ethnic disparities in NHPI and AA subgroups have been published using these data [18, 19]. In a recent analysis of PD patients using this dataset, we uncovered potential disparities for NHPI and Filipino patients with PD: among hospitalized PD patients, NHPI patients were youngest, followed by Filipino patients, compared to Whites, Japanese, and Chinese [20]. Medical comorbidities were higher in NHPI and Filipino patients.

One possible explanation for these disparities is increased medical comorbidities and decreased longevity in the NHPI and Filipino populations as a whole [21, 22]. Are NHPI and Filipino patients with PD more sick at younger ages because of these confounding population characteristics, or are there also unique biological differences between these groups? In order to answer this question, we determined the prevalence of hospitalized PD patients among total all case hospitalization in 5-year age increments and compared them across the racial/ethnic subgroups.

## METHODS

### Data source

The Hawaii statewide hospitalization registry database from 2016 to 2020 was utilized. The data were collected from all of Hawaii’s acute care hospitals [23], including hospitals located on the neighbor islands, by Laulima Data Alliance (LDA), a subsidiary of the Healthcare Association of Hawaii. LDA utilizes a Master Patient Index, which provides the ability to track individuals’ data over time and allows the evaluation of readmissions across all hospitals except for a hospital administrated by the US Army (which reports to a federal database). Unlike most hospitalization data registries of other states, the LDA differentiates NHPI from AA and categorizes AA into subgroups.

**Fig. 1:**
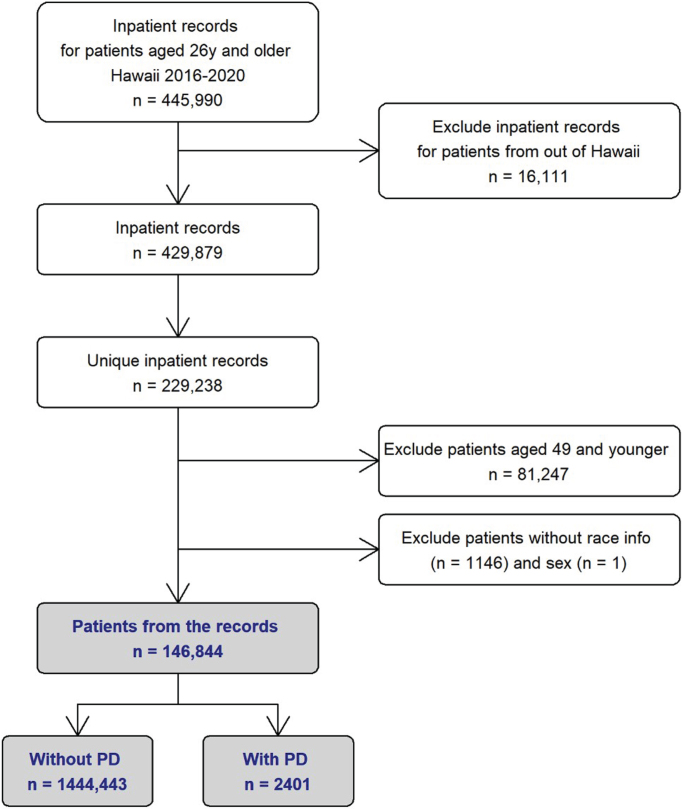
Flowchart Depicting Retrieval of Hospitalization Data from Hawaii Statewide Registry (2016–2020). PD, Parkinson’s disease.

Hospitalizations of adults aged≥26 years in Hawaii from January 2016 to December 2020 (the most recent available year at the time of data acquisition) were retrieved (*n* = 445,990 hospitalizations). Records were excluded for non-Hawaii residents (*n* = 16,111 hospitalizations). Individuals with multiple hospitalizations could complicate inferences of disparities. Using the LDA’s Master Patient Index, we identified 229,238 unique patients in the data set. Among the 229,238 unique patients, 2,419 patients were identified with a diagnosis of PD (ICD10 code G20) as their primary or secondary ICD10 code for hospitalization. Atypical parkinsonism, vascular parkinsonism and drug-induced parkinosnism all have different ICD10 codes and were excluded from this study. After initial analysis [20], we identified only 18 patients with PD who were 50 years or younger. For the race/ethnic subgroup comparison to be meaningful from statistical point of view, we decided to include only patients who were 50 years or older at their last hospitalization. This resulted in a total of 146,844 hospitalized patients and 2,401 PD patients (Fig. 1).

### Main measures

We obtained the following data: age in 5-year increments: sex, county of residence, primary source of payment, discharge status, length of stay, Charlson Comorbidity Index, and presence of Diabetes Mellitus (for comparison analysis, which is described in the discussion section). When individuals had multiple hospitalizations, characteristics at the last hospitalization were used for analyses.

### Race/Ethnicity

The LDA race/ethnicity variable was created from race/ethnicity categories available consistently across all hospitals in Hawaii. Race/Ethnicity data are typically provided by patient self-report at registration and include only one primary race. Mixed race individuals are categorized by their primary self-reported racial/ethnic identity. In this analysis, patients’ race was categorized into the five most common race categories: White, Japanese, Filipino, Chinese, NHPI, and the rest as “Other”. Other categories included Black, American Indian, Alaska Native, and other Asian, such as Korean, Vietnamese, and Indians. We calculated the racial composition by age subgroups of the entire Hawaii population determined based on Census data [24, 25].

**Table 1:** Demographic Characteristics of Patient without and with Parkinson’s Disease: Hawaii State Registry (2016–2020)

Without PD	With PD
	*n* = 144,443 (98.4%)	*n* = 2401 (1.6%)	*p* ^a^
**Race/Ethnicity, *n* (%)**			< 0.001
White	41,691 (98.3)	728 (1.7)
Filipino	22,833 (98.8)	268 (1.2)
Japanese	31,841 (97.7)	758 (2.3)
Chinese	8235 (97.7)	193 (2.3)
NHPI	25,272 (99.1)	229 (0.9)
Other	14,571 (98.5)	225 (1.5)
**Age in year by race/ethnicity, mean (SD)**
White	69.7 (11.2)	77.3 (8.9)	< 0.001
Filipino	71.0 (12.0)	78.4 (9.5)	< 0.001
Japanese	77.1 (12.9)	81.4 (9.3)	< 0.001
Chinese	75.6 (13.0)	81.2 (9.7)	< 0.001
NHPI	66.2 (10.8)	74.2 (9.9)	< 0.001
Other	69.6 (12.1)	77.6 (9.9)	< 0.001
**Age group (y)^b^, *n* (%)**			< 0.001
50–54	13,067 (99.8)	22 (0.2)
55–59	16,337 (99.6)	59 (0.4)
60–64	19,045 (99.4)	117 (0.6)
65–69	20,671 (98.9)	230 (1.1)
70–74	19,745 (98.3)	333 (1.7)
75–79	16,153 (98.4)	430 (2.6)
80–84	13,244 (96.6)	460 (3.4)
+85	26,181 (97.2)	750 (2.8)
**Sex, *n* (%)**			< 0.001
Male	74,497 (98.1)	1434 (1.9)
Female	69,946 (98.6)	967 (1.4)
**County of residence^b^, *n* (%)**			< 0.001
Honolulu	97,351 (98.2)	1820 (1.8)
Hawaii	24,052 (98.9)	267 (1.1)
Maui	15,801 (98.7)	209 (1.3)
Kauai	7239 (98.6)	105 (1.4)
**Primary source of payment^b,c^, *n* (%)**			< 0.001
Medicare	89,185 (97.7)	2,119 (2.3)
Medicaid	15,581 (99.6)	60 (0.4)
Private	34,545 (99.5)	160 (0.5)
Self-pay	1193 (99.3)	9 (0.7)
Other	3889 (98.7)	53 (1.3)
**Discharge status^b^, *n* (%)**			< 0.001
Home	92,646 (99.2)	790 (0.8)
Skilled Nursing	16,389 (96.4)	611 (3.6)
Home care service	10,765 (97.9)	235 (2.1)
Expired	12,116 (97.6)	295 (2.4)
Other	12,527 (96.4)	470 (3.6)
**Length of stay^b^, median (IQR)**			< 0.001
	4 (2, 7)	5 (3, 8)
**In-hospital expiration^d^, *n* (%)**			< 0.001
	12,117 (8.4)	295 (12.3)

PD, Parkinson’s disease. ^a^*p*-values were for comparing patients with and without PD diagnosis by Welch two sample *t*-test or Wilcoxon rank sum test for continuous variables and Pearson’s Chi-squared tests or Fisher’s exact tests for categorical variables. ^b^At the last hospitalization during the study period. ^c^Exclude those with missing information (0.03% of total). ^d^Expired during the study period.

### Statistical analysis

Patients’ characteristics were summarized with means and standard deviations (SDs) or medians and interquartile ranges (IQRs) for continuous variables and frequencies and percentages for categorical variables. Differences in continuous variables between groups were examined by Welch Two sample *t*-tests or Wilcoxon rank sum tests. Differences in categorical variables between or across groups were examined by Pearson’s chi-squared tests or Fisher’s exact tests. A *p*-value less than 0.05 was considered statistically significant. All statistical analyses were performed using R package version 4.2.1.

## RESULTS

During the study period, there were a total of 146,844 patients aged 50 years and older hospitalized in Hawaii. Among them, 2,401 (1.6%) patients had a diagnosis of PD.

Table 1 summarizes the demographic and clinical characteristics of all hospitalization cases in the state of Hawaii during the study period. Japanese and Chinese had the highest prevalence of hospitalized PD patients (both 2.3%) among all cases, and NHPI had the lowest (0.9%) of hospitalized PD patients. Compared to non-PD hospitalized patients, patients with PD were older in all race groups. The prevalence of hospitalized PD patients increased with age. There were more males, Honolulu County residents, and Medicare beneficiaries for the PD group. Inpatient expiration rate and discharge status to skilled nursing facilities were higher in the PD group. Median length of stay was higher in the PD group. The most common diagnosis code for discharge primary diagnosis was A419: Sepsis, unspecified organism, followed by J690: Pneumonitis due to inhalation of food and vomit for all race/ethnic subgroups.

**Fig. 2:**
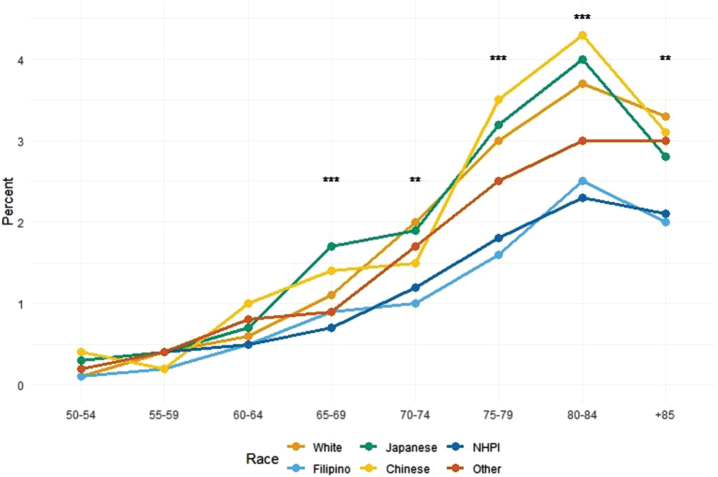
Prevalence of Hospitalized Parkinson’s Disease Patients among All Hospitalization Cases for Each Racial/Ethnic Subgroup and for Each 5-Year Age Increments: Hawaii State Registry (2016–2020). NHPI, Native Hawaiian and Pacific Islander. The asterisks denote statistical significance found in the Chi-squared test or Fisher’s exact test to examine differences in proportions across the racial/ethnic groups in the age group (**p < 0.01, ***p < 0.001).

The prevalence of hospitalized PD patients among all hospitalization cases by race/ethnic subgroups and by 5-year-age groups is shown in Fig. 2 (see Supplementary Table 1 for further detail). In all race/ethnic subgroups, the prevalence of hospitalized PD patients increased with increasing age. The plot over age for Whites, Japanese, and Chinese were almost identical, with the prevalence of hospitalized PD patients among all total case hospitalization reaching approximately 4% at age 80–84 years old. While the prevalence of hospitalized PD patients also increased with increasing age, NHPI/Filipinos had a different prevalence plot over age, where the prevalence of hospitalized PD patients reaching no higher than 2.5% at age 80–84 year-old. There was male predominance in all race/ethnic subgroups: most notable in Whites and NHPIs. Whites had the lowest in-hospital expiration rate (Supplementary Table 2).

**Fig. 3:**
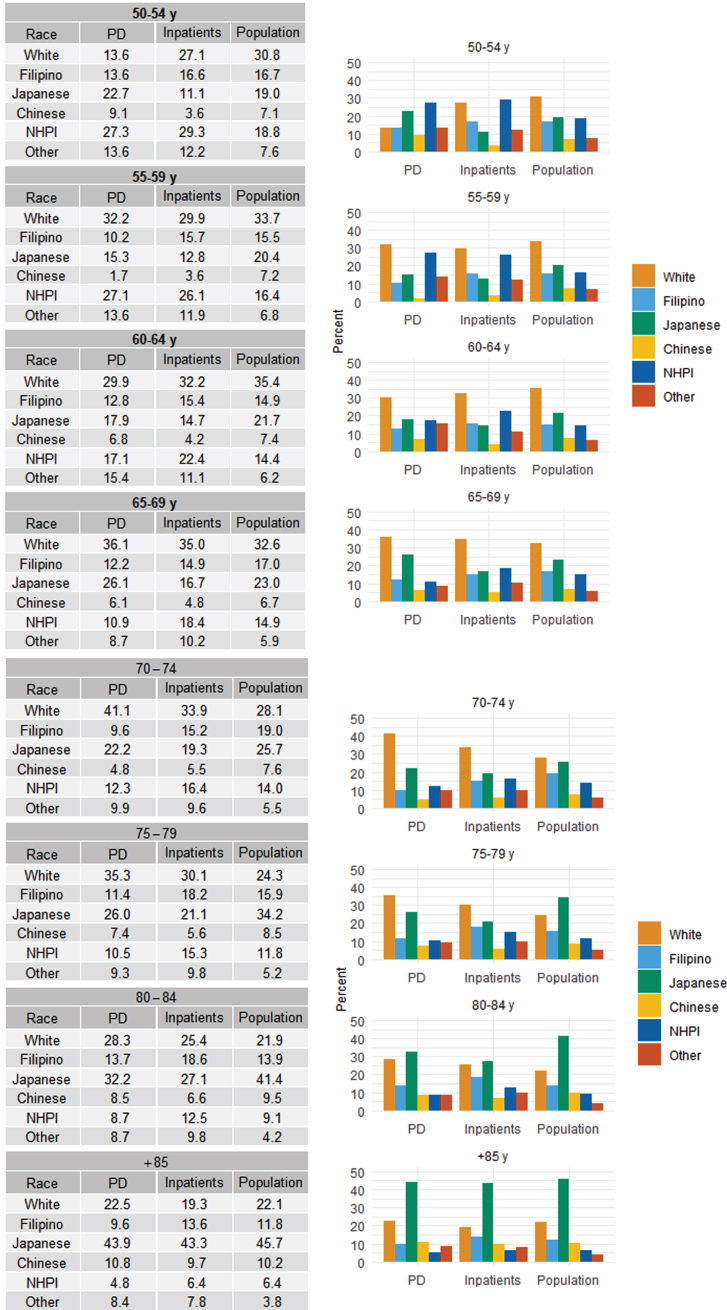
Racial/Ethnic Composition of Hospitalized Parkinson’s Disease, All Hospitalized Patients, and Hawaii General Population in 8 Age Subgroups^a^: Hawaii State Registry (2016–2020). PD, Parkinson’s disease; NHPI, Native Hawaiian and other Pacific Islander. ^a^ Percentages of hospitalized patients with Parkinson’s disease (PD), all hospitalized patients (Inpatients), and the Hawaii general population based on Census single race estimates (Population) (references 24, 25), by race/ethnicity groups.

Figure 3 shows the racial/ethnic subgroup composition of PD patients (PD), hospitalization cases (Inpatients), and the estimated Hawaii population (Population) [23, 24] for the eight age subgroups. The racial compositions in hospitalization cases (Inpatients) were overall similar to those of the general populations: Japanese and Chinese tended to be hospitalized less compared to those of their respective populations, and NHPI tended to be hospitalized more compared to the population in all age subgroups.

Table 2 summarizes the Charlson Comorbidity Index (CCI) scores for PD vs. non-PD hospitalization for each race/ethnic subgroup. Except for Whites and Other, there was no statistically significant difference in the number of CCI scores between patients with PD and without PD. NHPI and Filipino had the highest percent of patients with a CCI score greater than 2, regardless of PD status.

**Table 2:** Distributions of Parkinson’s Disease Status by Charlson Comorbidity Index Score among 6 Race/Ethnicity Groups^a^: Hawaii State Registry (2016–2020)

White			Filipino		
CCI, *n* (%)	Without PD	With PD	*p* ^b^	Without PD	With PD	*p* ^b^
	*n* = 41,691 (98.3%)	*n* = 728 (1.7%)		*n* = 22,833 (98.8%)	*n* = 268 (1.2%)
0	13,171 (31.6)	185 (25.4)	0.005	3726 (16.3)	31 (11.6)	0.070
1	11,996 (28.8)	225 (30.9)		5963 (26.1)	65 (24.3)
2	8317 (19.9)	158 (21.7)		5440 (23.8)	65 (24.3)
> 2	8207 (19.7)	160 (22.0)		7704 (33.7)	107 (39.9)
Japanese			Chinese		
CCI, *n* (%)	Without PD	With PD	*p* ^b^	Without PD	With PD	*p* ^b^
	*n* = 31,841 (97.7%)	*n* = 758 (2.3%)		*n* = 8,235 (97.7%)	*n* = 193 (2.3%)
0	6087 (19.1)	150 (19.8)	0.11	1576 (19.1)	43 (22.3)	0.50
1	9172 (28.8)	244 (32.2)		2254 (27.4)	51 (26.4)
2	7576 (23.8)	174 (23.0)		1830 (22.2)	47 (24.4)
> 2	9006 (28.3)	190 (25.1)		2575 (31.3)	52 (26.9)
NHPI			Other		
CCI, *n* (%)	Without PD	With PD	*p* ^b^	Without PD	With PD	*p* ^b^
	*n* = 25,272 (99.1%)	*n* = 229 (0.9%)		*n* = 14,571 (98.5%)	*n* = 225 (1.5%)
0	3755 (14.9)	22 (9.6)	0.086	3305 (22.7)	33 (14.7)	0.009
1	5868 (23.2)	53 (23.1)		3847 (26.4)	54 (24.0)
2	5757 (22.8)	50 (21.8)		3198 (21.9)	61 (27.1)
> 2	9892 (39.1)	104 (45.4)		4221 (29.0)	77 (34.2)

PD, Parkinson’s disease; CCI, Charlson comorbidity index. ^a^Numbers are column percentage of PD status and CCI scores based on the diagnoses patients had at the last hospitalization during the study period. ^b^A *p*-value was obtained by Pearson’s Chi-squared test.

## DISCUSSION

Our study found that the overall prevalence of hospitalized PD patients was 1.6% of total all case hospitalization over age 50 years old in the state of Hawaii from 2016 to 2020. Japanese and Chinese had a higher prevalence of hospitalized PD patients (2.3%), followed by 1.7% for White and 1.2% for Filipinos. NHPI had the lowest prevalence of hospitalized PD patients: 0.9%.

Prevalence of hospitalized PD patients among total all case hospitalization increased with age, reaching approximately 4% at age 80–84 years old for White, Japanese and Chinese patients, but only 2.5% for the NHPI and Filipino groups. As reported previously, PD was more common in male for all racial/subgroups.

Because our study is limited to hospitalized patients, we cannot generalize our results to overall prevalence of PD in our population. Nevertheless, our study raises the possibility of differences and disparity among different race/ethnic subgroups that warrant further investigation.

Previous studies attempting to understand if prevalence of PD varies by age, by sex, and by geographic region have acknowledged the analytical challenges: different studies using different methodologies and criteria for PD diagnosis due to lack of biomarker [26, 27]. Despite limitation, previous epidemiological studies have consistently shown that PD is more common in men and increases steadily with aging in people over 65 years-old; PD prevalence was highest in people over 80 years-old [28]. The difference in prevalence among different races and geographical regions has been less consistent. One meta-analysis noted higher prevalence in Northern America, Europe, and Australia, compared to Asia only in individuals age 70–79 years old [26] whereas another study showed East Asia to have the highest PD prevalence in 2019, and Oceania region to have the lowest prevalence [28]. Another study showed that PD prevalence was higher in countries with higher Socio-demographic Index (SDI: a compound measure of income per capita, education, and fertility). Possible explanation for this association included difference in rate of exposure to potential risk factors, such as, pesticides, solvents, or metals. Alternative explanations included better ascertainment of PD in higher SDI countries through better study methodology, better awareness of PD, or increased access to healthcare [29].

In our study, there was slight male predominance of PD, and the 6 to 4 ratio is consistent with previous epidemiological studies. Male predominance was stronger in White and NHPI groups. This is also consistent with previous reports that male predominance is not as notable in Asians with male to female ratio ranging from 0.95–1.2 [30]. We do need to be cautious about interpreting hospitalized dataset: men and women may be hospitalized for different reasons, resulting in different rates of hospitalization.

The racial composition of the total all case hospitalization was relatively similar to that of the population estimate in each age group (Fig. 3), indicating that the total all case hospitalization (inpatient) group was a good representation of the general population. In another attempt to check the quality of our data/methodology, we analyzed the prevalence of hospitalized diabetes mellitus (DM) patients in this dataset and compared it to the known previously published population estimate of DM prevalence among different race and age groups [31]. Despite differences in methodology (our dataset consists only of inpatients whereas the published DM data was ascertained from the general population, and age range for the youngest group in our dataset was 50–54 where as it was 45–54 in the published DM data), the overall pattern of hospitalized DM patients in our data was relatively similar to the published population data: Filipinos and NHPI had the highest prevalence of DM, followed by Chinese/Japanese then Whites (Supplementary Figure 1).

In our study, the prevalence of hospitalized PD patients was lowest in NHPI. There were two distinct patterns of prevalence as patients aged. Whites, Chinese and Japanese followed an identical pattern. Filipino/NHPI had a different pattern, not quite reaching the same prevalence of hospitalized of PD patients among total all case hospitalization as they aged.

The reason for this difference is unclear. One of the possible explanations for these disparities were increased medical comorbidity and decreased longevity in NHPI and Filipino population. Native Hawaiians are known to have higher rates of cardiovascular comorbidities, obesity and diabetes [10], and present with stroke at an earlier age [8]. Similar health disparities in cardiovascular health have been observed in Filipinos compared to other Asian subgroups. Japanese tend to have strong health profiles compared to other racial/ethnic groups in Hawaii and have a longer life expectancy than most racial groups [22]. In our study, there was no difference in CCI between PD and non PD group for each race/ethnic subgroups, but NHPI and Filipino patients, as a group, had a higher proportion of having a CCI over 2 (Table 2), indicating that they are overall sicker and may lead to decreased longevity. In-hospital expiration rate for PD patients was lowest for White patients in our study. Japanese/Chinese patients with PD were older, so this may explain their higher in-hospital expiration (compared to Whites), whereas for Filipino/NHPI patients, a higher in-hospital expiration rate (compared to Whites) may yet be another indication for health disparity. The racial composition does change with aging, and there are more Japanese and less NHPIs and Filipinos in both the total all case hospitalization as well as population estimate in the 75 years or older groups. However, even accounting for the population difference by using the total all case hospitalization as the denominator, PD prevalence was lower in NHPIs and Filipinos among people over age 75-years old compared to Whites, Japanese, and Chinese.

Genetic/biological differences may account for differences in prevalence, age of onset and disease course for PD among different races. Genome-wide association studies have identified similarities and differences in genetic risk factors between Asian and European individuals for PD [32]. Risk gene loci that can affect age of onset [33] or disease course [34] have been identified in different populations. PINK1, a known genetic locus for young onset PD is identified in Filipinos and Polynesians [35, 36], but beyond that, studies analyzing PD polygenic risk scores in NHPIs or Filipinos are lacking. The importance of studying genetics in underserved and diverse population have been identified as future goals [37].

Lack of access to healthcare and cultural differences including knowledge/awareness of disease can result in under diagnosis. Previous studies showed that African Americans and Chinese Americans are more likely to perceive PD symptoms as a normal part of aging than White-Americans, raising the possibility of underdiagnoses and underutilization of medical resources [38]. Lack of access to healthcare, such as medication, advanced therapies [39], as well as ancillary therapy (physical therapy, speech therapy) [40] can lead to more rapid and malignant disease course in NHPI and Filipino population. Medical comorbidities can aggravate overall health outcomes as discussed above. Other possible explanations include differences in environmental factors, such as cigarette smoking or exposure to pesticides. Inverse relationship between smoking and prevalence of PD has been documented [41]. NHPIs have one of the highest smoking rates in Hawaii [42] and another study reported Filipinos had a higher smoking rates compared to other Asian ethnic groups [43]. Pesticides were commonly used on pineapple plantations in Hawaii and data from the prospective Kuakini Honolulu-Asia Aging Study indicate an association of working on a plantation with higher PD risk in a cohort of Japanese American men [44]. However, pesticides were also extensively used as termiticides, so essentially all residents in the state would have been exposed [45]. Administrative error, for example, coding omission (coding fatigue due to multiple diagnosis code, disproportionately affecting NHPI and Filipino with higher medical comorbidities) is another possible explanation.

Our study has several limitations. First, we were only able to analyze retrospectively factors present in the hospitalization data and the results may be subject to unmeasurable bias. PD is a chronic disease typically cared for in outpatient settings. There are many important factors associated with PD and its treatment, such as clinical phenotype, disease duration, and medication regimen, which are not available in our data: thus, the interpretation and conclusion that can be drawn from our study is limited. Secondly, for patients with multiple hospitalizations we analyzed the last hospitalizations, which may subject to unmeasurable bias. Third, our analysis only represents 5 years so the age of hospitalization is arbitrary and subject to unmeasurable bias. Fourth, the hospitalization claims data are known to be subject to significant diagnostic misclassification or diagnostic errors and not always accurate. The study did not assess whether the patients met the diagnostic criteria for PD and relied on the diagnoses made in uncontrolled outpatient or inpatient settings. Lastly, this study is limited to Hawaii and the results are not generalizable.

Despite these limitations, our results indicate that there may be important differences and disparities for NHPI and Filipino, two understudied groups in PD research. Further studies, including genetic studies as well as prospective cohort studies, are urgently needed to understand the biological and socioeconomic factors that are driving the observed disparities. MM and JJC were partially supported by the U54MD007601, and MM was also partially supported by the U54GM138062 from the National Institute of Health (NIH). The content is solely the responsibility of the authors and does not necessarily represent the official views of NIH or the US government.

## Supplementary Material

Supplementary Material

## Data Availability

The data utilized in the report was under the Data Use Agreement with Laulima Data Alliance (LDA), a non-profit subsidiary of the Healthcare Association of Hawaii. The use and sharing of the limited data set requires a review and approval of LDA. The statistic software (R) is a free software environment for statistical computing and graphics.
