# Supplementary material for: The Prevalence of Hospitalized Parkinson’s Disease Patients in All Case Hospitalization among Different Race/Ethnic Subgroups in Hawaii
Source: J Parkinsons Dis. 2024 Jun 4;14(4):725–35. doi: 10.3233/JPD-230341 (PMC11191512; doi:10.3233/JPD-230341)
Supplement: Supplementary Material [file jpd-14-jpd230341-s001.pdf]

Supplementary Material

The Prevalence of Hospitalized Parkinson's Disease Patients in All Case Hospitalization among Different Race/Ethnic Subgroups in Hawai'i

Supplementary Table 1. Prevalence of Hospitalized Parkinson's Disease patients among Total All Case Hospitalization for Each Racial/Ethnic Subgroup for 5-Year Age Increments

	White with PD n=728 (1.7%)	Filipino with PD n=268 (1.2%)	Japanese with PD n=758 (2.3%)	Chinese with PD n=193 (2.3%)	NHPI with PD n=229 (0.9%)	Other with PD n=225 (1.5%)
Age, n (%)[a]						
50-54 y	3 (0.1)	3 (0.1)	5 (0.3)	2 (0.4)	6 (0.2)	3 (0.2)
55-59 y	19 (0.4)	6 (0.2)	9 (0.4)	1 (0.2)	16 (0.4)	8 (0.4)
60-64 y	35 (0.6)	15 (0.5)	21 (0.7)	8 (1.0)	20 (0.5)	18 (0.8)
65-69 y	83 (1.1)	28 (0.9)	60 (1.7)	14 (1.4)	25 (0.7)	20 (0.9)
70-74 y	137 (2.0)	32 (1.0)	74 (1.9)	16 (1.5)	41 (1.2)	33 (1.7)
75-79 y	152 (3.0)	49 (1.6)	112 (3.2)	32 (3.5)	45 (1.8)	40 (2.5)
80-84 y	130 (3.7)	63 (2.5)	148 (4.0)	39 (4.3)	40 (2.3)	40 (3.0)
+85 y	169 (3.3)	72 (2.0)	329 (2.8)	81 (3.1)	36 (2.1)	63 (3.0)

PD: Parkinson's Disease. [a] Numbers are counts of hospitalized patients with PD and percentages of all hospitalized patients for the age/race group. The chi-squared test to examine differences in the proportions of PD status groups across the age groups found a statistical significance in all race/ethnicity groups (*ps*<0.001). Fisher's exact test to examine differences in the proportions of age groups across the race groups found a statistical significance (*p*<0.001).

Supplementary Table 2. Male to Female Proportions and In-hospital Mortality of Parkinson's Disease Patients for Each Racial/Ethnic Subgroup

	White with PD n=728	Filipino with PD n=268	Japanese with PD n=758	Chinese with PD n=193	NHPI with PD n=229	Other with PD n=225	*p*[a]
Sex, n (%)							
Male	486 (66.8)	156 (58.2)	418 (55.1)	110 (57.0)	145 (63.3)	119 (52.9)	<0.001
Female	242 (33.2)	112 (41.8)	340 (44.9)	83 (43.0)	84 (36.7)	106 (47.1)	
In-hospital mortality, n (%)							
Expired	65 (8.9)	39 (14.6)	96 (12.7)	26 (13.5)	34 (14.8)	35 (15.6)	0.025
Not expired	663 (91.1)	229 (85.4)	662 (87.3)	167 (86.5)	195 (85.2)	190 (84.4)	

PD, Parkinson's disease. [a] p-values were obtained from the chi-squared test.

Supplementary Figure 1. A) Proportions of hospitalized patients with diabetes mellitus by race group: Hawaii State Registry (2016-2020). B) Diabetes prevalence estimated from Hawai'i Behavioral Risk Factor Surveillance System, 2011, 2013, 2015. Adapted from Uchima et al. (2019) *Prev Chronic Dis* **16**, 180187.

A

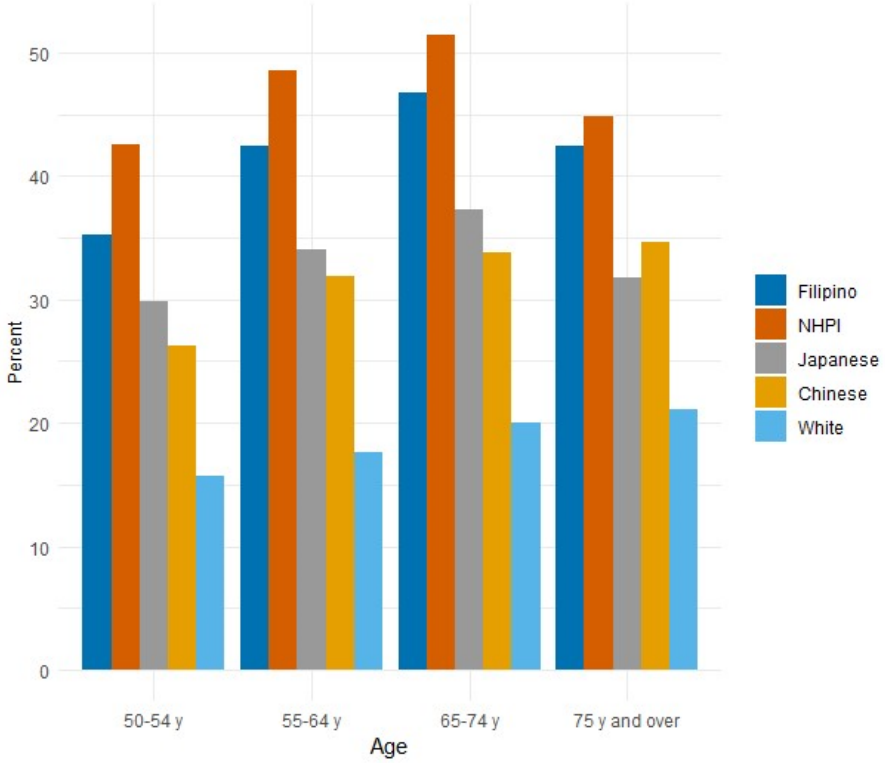

B

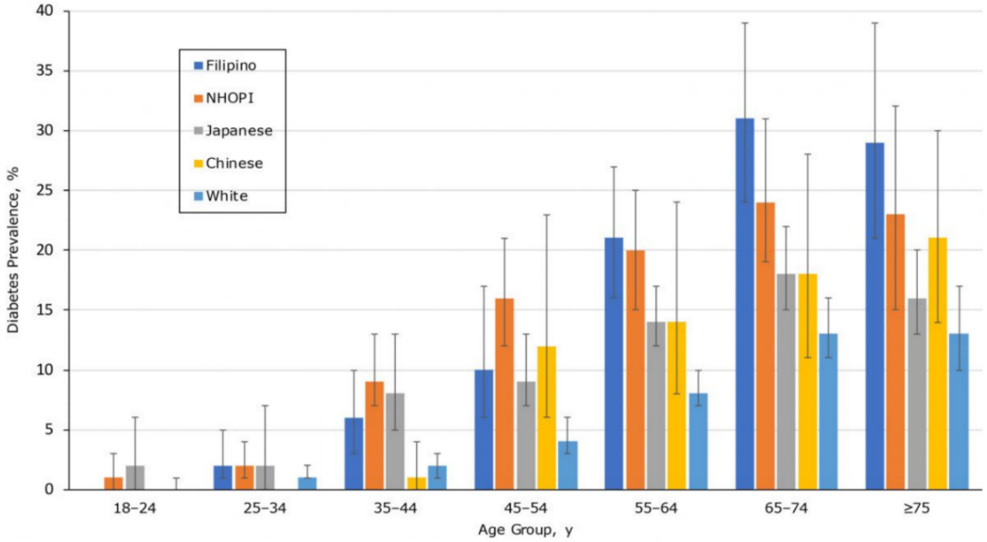

NHPI/NHOPI, Native Hawaiian and other Pacific Islander
